# Sympathy

**Published:** 1909-06

**Authors:** Victoria A. H. Duggan


For Texas Medical Journal.

### Sympathy.

Sympathy is the touchstone of the heart
It is the spring from which the tear-drops start,
Like balm, which from the wound removes the smart.

It is a zephyr from off friendship's plain
It fans the coals of heart life into flame,
And eases what would otherwise be pain!

It is the keystone to a noble arch
Which rears its masonry, within the heart;
Thus Sympathy, and Friendship, ne'er can part!

VICTORIA A. H. DUGGAN.

